# Smn-Deficiency Increases the Intrinsic Excitability of Motoneurons

**DOI:** 10.3389/fncel.2017.00269

**Published:** 2017-09-05

**Authors:** Saravanan Arumugam, Ana Garcera, Rosa M. Soler, Lucía Tabares

**Affiliations:** ^1^Department of Medical Physiology and Biophysics, School of Medicine University of Seville Seville, Spain; ^2^Unitat de Senyalització Neuronal, Departament de Medicina Experimental, Universitat de Lleida-IRBLLEIDA Lleida, Spain

**Keywords:** spinal muscular atrophy (SMA), motoneurons, hyperexcitability, ion currents, synapses

## Abstract

During development, motoneurons experience significant changes in their size and in the number and strength of connections that they receive, which requires adaptive changes in their passive and active electrical properties. Even after reaching maturity, motoneurons continue to adjust their intrinsic excitability and synaptic activity for proper functioning of the sensorimotor circuit in accordance with physiological demands. Likewise, if some elements of the circuit become dysfunctional, the system tries to compensate for the alterations to maintain appropriate function. In Spinal Muscular Atrophy (SMA), a severe motor disease, spinal motoneurons receive less excitation from glutamatergic sensory fibers and interneurons and are electrically hyperexcitable. Currently, the origin and relationship among these alterations are not completely established. In this study, we investigated whether Survival of Motor Neuron (SMN), the ubiquitous protein defective in SMA, regulates the excitability of motoneurons before and after the establishment of the synaptic contacts. To this end, we performed patch-clamp recordings in embryonic spinal motoneurons forming complex synaptic networks in primary cultures, and in differentiated NSC-34 motoneuron-like cells in the absence of synaptic contacts. Our results show that in both conditions, Smn-deficient cells displayed lower action potential threshold, greater action potential amplitudes, and larger density of voltage-dependent sodium currents than cells with normal Smn-levels. These results indicate that Smn participates in the regulation of the cell-autonomous excitability of motoneurons at an early stage of development. This finding may contribute to a better understanding of motoneuron excitability in SMA during the development of the disease.

## Introduction

Spinal muscular atrophy (SMA), the leading genetic cause of infant mortality (Sugarman et al., 2012), is an autosomal recessive monogenic disease characterized by neuromuscular dysfunction, loss of motoneurons and paralysis. It is caused by reduced levels of the survival of motor neuron protein (SMN; Lefebvre et al., 1995, 1997; Crawford and Pardo, 1996), which is a ubiquitous multifunctional protein that localizes to the cell nucleus and soma and participates in the assembly of the splicing machinery (Gubitz et al., 2004; Battle et al., 2006). During embryonic development and postnatal maturation, SMN is also present in dendrites and axons where is involved in the transport of mRNA for local translation (Rossoll et al., 2003; Rossoll and Bassell, 2009; Donlin-Asp et al., 2016; Fallini et al., 2016).

In SMA mouse models, Smn deficiency produces characteristic functional and structural alterations in sensorimotor neural circuits. Particularly significant are deficient neurotransmitter release at motor nerve terminals (Kong et al., 2009; Ruiz et al., 2010; Torres-Benito et al., 2011; Ruiz and Tabares, 2014; Tejero et al., 2016), reduced numbers of both proprioceptive sensory inputs (Ling et al., 2010; Park et al., 2010; Mentis et al., 2011), and interneuron inputs (Simon et al., 2016), and hyperexcitability of motoneurons (Mentis et al., 2011; Liu et al., 2015). Currently, the origin and relationship among these alterations are not completely established. The loss of synaptic inputs onto motoneurons could result from the intrinsic alterations in the motoneurons themselves, or from impairments in the cells interacting with the motoneurons (sensory neurons, interneurons, astrocytes and microglia) In favor of a cell-autonomous (intrinsic) mechanism is the observation that the central and peripheral synaptic defects are reproduced by depleting Smn only in motoneurons (Park et al., 2010; McGovern et al., 2015). Moreover, selective restoration of Smn within motoneurons in an SMA mouse model significantly improves the motor phenotype, including the normalization of the synaptic inputs (Gogliotti et al., 2012; Martinez et al., 2012; Arnold et al., 2016). However, recently, in a series of elegant experiments in differentiated mouse embryonic stem cells, it has been shown that the reduction in Smn levels only in interneurons decreases the number of inputs onto wild-type motoneurons, suggesting a non-cell autonomous origin (Simon et al., 2016). In addition, reduced expression of Smn in non-neuronal cells may also contribute to the pathogenesis of SMA; for example, the interaction between astrocytes and motoneurons, which is known to contribute to formation and maintenance of synaptic contacts, are diminished in SMA mouse models (Rindt et al., 2015; Zhou et al., 2016).

The origin of motoneuron electrical hyperexcitability in SMA is still under analysis. Normally, spinal motoneurons are excitable before synaptogenesis. In rodents, synaptogenesis begins during the last week of gestation and continues during the first postnatal weeks. During this period, the passive and active electrical membrane properties vary, action potential threshold progressively shifts to more negative values and ion channel expression increases. These changes result in a rise in excitability, functionally relevant to the transduction of the growing neural activity into more complex locomotor activity and to the maturation of the neuromuscular junction (NMJ) and the muscle apparatus. Mature neurons are also able to adjust their intrinsic excitability and the number and strength of their synaptic contacts in response to the functional demand (Desai, 2003; Davis, 2006; Marder and Goaillard, 2006). Likewise, pathological processes that alter the synaptic drive can lead to changes in the passive properties and the voltage-dependent membrane ionic conductances of neurons (Desai et al., 1999; Aptowicz et al., 2004). Therefore, hyperexcitability could be a homeostatic response to the reduction in presynaptic contacts. In fact, the input resistance is increased in wild-type motoneurons synapsing with Smn-deficient interneurons (Simon et al., 2016). Also, *in vivo*, the deficit in transmitter release at the NMJ could be a factor to the rise in excitability in motoneurons (Nick and Ribera, 2000; Nakanishi et al., 2005). Here, we investigated whether Smn-deficiency alters mouse motoneuron excitability and voltage-dependent ion channel density in the presence and the absence of synaptic contacts. Our results suggest that the intrinsic excitability of motoneurons is regulated cell autonomously by Smn at an early stage of development and that Smn participates not only in the establishment and maintenance of synaptic contacts but also in the regulation of the intrinsic excitability of the motoneurons.

## Materials and Methods

### Motoneuron Isolation and Culture

Spinal motoneurons were obtained from dissected ventral spinal cords of E12.5 FVB mice as described before (Gou-Fabregas et al., 2009). Briefly, the dissected ventral cords were chopped, rinsed in GHEBS buffer (137 mM NaCl, 2.7 mM KCl, 22.2 mM glucose, 25 mM HEPES pH 7.4, and 20 IU/ml penicillin plus 20 mg/ml streptomycin) and incubated in trypsin (Sigma; final concentration 0.025%) for 10 min at 37°C. Trypsinization was followed by mechanical dissociation. Cells, dissociated by pipetting through a Gilson blue cone in complete culture medium (Leibovitz’s 15 (L15) medium supplemented with 18 mM glucose, 2.5 mM glutamine, 10% heat-inactivated horse serum and 20 U/ml penicillin plus 20 μg/ml streptomycin) were centrifuged (5 min at 140× *g*) and collected under a 4% bovine serum albumin (BSA) cushion. The density gradient was prepared by using freshly prepared Iodixanol (OptiPrep, Axis-Shield) solution (12.5%) in GHEBS. The largest cells were isolated by centrifugation (10 min at 520× *g*) on this gradient. The collected cells were pooled in a tube, and then plated over cover slips polyornithine/laminin-coated (19 mm) in 12-well plates (50,000 cells/well). Neurobasal (Invitrogen) culture medium was supplemented with B27 (Invitrogen), horse serum (2% v/v), L-glutamine (0.5 mM), and 2-mercaptoethanol (25 μM). The culture medium was further supplemented with a cocktail of recombinant neurotrophic factors (1 ng/ml brain-derived neurotrophic factor, 10 ng/ml glial cell line-derived neurotrophic factor, 10 ng/ml cardiotrophin-1 and 10 ng/ml hepatocyte growth factor; Peprotech). For long-term experiments, the culture medium was changed every 4–5 days and the purified motoneurons culture were maintained under standard cell culture conditions of 5% CO_2_ at 37°C. Aphidicolin (Sigma-Aldrich), which is an antimitotic agent and DNA polymerase inhibitor, was added at a final concentration of 1.5 μg/mL to the culture medium supplemented with neurotropic factors. All experiments were performed according to the guidelines of the European Council Directive for the Care of Laboratory Animals and approved by the Ethics committee for Animal Experimentation of the Junta de Andalucía (ref. 7-1-15-173).

### NSC-34 Cell Line Culture

NSC-34 cells, a hybrid cell line produced by fusion of enriched embryonic mouse spinal motoneurons with mouse neuroblastoma cells (Cashman et al., 1992), were cultured and maintained in Dulbecco’s modified Eagle Medium (DMEM) 1× (4.5 g/L glucose), supplemented with 10% fetal bovine serum (FBS), 20 U/ml penicillin plus 20 μg/ml streptomycin and 2 mM glutamine. The cells were grown in an atmosphere of 5% CO_2_ at 37°C in 100 mm tissue culture dishes. The cell cultures were split at about 80% confluence. For the experiments, 60,000 cells/well, at passages 12–16, were seeded on laminin coated coverslips (19 mm) in 12-well plates. After 2 h, the media was changed to DMEM 1× with 1% FBS to induce differentiation and transduced with shSmn or Empty Vector (EV) lentiviral constructs. Media was changed every 24 h. Cells were used for experiments 72 h after lentiviral interference.

### Plasmids and Production of Lentiviral Particles

For preparing the lentiviral constructs, we used the second-generation lentiviral system, which comprises three plasmids (Transfer (vector), Packaging and envelope). The constructs were generated in pSUPER.retro.puro (OligoEngine, Seattle, WA, USA) using specific oligonucleotides (Invitrogen) targeting Smn sequence (shSmn forward: gatccccAGTAAAGCACACAGCAAGTttcaagagaACTTGCTGTGTGCTTTACTttttt and reverse: agctaaaaaAGTAAAGCACACAGCAAGTtctcttgaaACTTGCTGTGTGCTTTACTggg. Adaptors to clone the oligonucleotides into the BglII/HindIII sites of pSUPER.retro.puro were added as required. Lentiviral constructs were generated by digesting pSUPER-sh with EcoRI and ClaI to replace the H1 promoter with the H1-short hairpin RNA (shRNA) cassette in pLVTHM. The pLVTHM vector contains the Green Fluorescent Protein (GFP) under the control of an EF-1 alpha promoter for monitoring transduction efficiency. pLVTHM, pSPAX2 and pMD2G were kindly provided by Dr. Trono (University of Geneva, Switzerland). Lentiviruses were propagated in human embryonic kidney 293T (HEK293T) cells using the polyethylenimine (Sigma) cell transfection method. Twenty micrograms of pLVTHM-Smn or pLVTHM-EV, 13 μg of pSPAX2, and 7 μg of pM2G were transfected to HEK293T cultures. Cells were allowed to produce lentivirus for 4 days. Then the medium was centrifuged at 1200× *g* for 5 min, and the supernatant was filtered using a 45 μm pore diameter filter. The medium containing the lentiviruses was stored at 4°C. Biological titers of the viral preparations, expressed as the number of transducing units per mL (TU/mL), were determined by transducing HEK293T cells in limiting dilutions. After 48 h the percentage of GFP positive cells was measured, and viruses at 4 × 10^5^− 1 × 10^6^ TU/mL were used for the experiments. For lentiviral transduction, cells were incubated with the medium containing lentivirus (2 TU/ cell) after 2 h of plating. The medium was changed 20 h later, and transduction efficiency was monitored in each experiment by direct counting GFP-positive cells. RNA interference efficiency was monitored by western blot analysis using an anti-SMN antibody.

### Drugs

DL(-)-2-amino-5-phosphonopentanoic acid (APV), a selective NMDA receptor antagonist, and 6-cyano-7-nitroquinoxaline-2,3-dione (CNQX), an AMPA/kainate receptor antagonist, were purchased from Sigma-Aldrich. Media containing 100 μM APV and 20 μM CNQX was given to the cells after plating. The media was replaced every day. Final electrophysiological recordings of the treated versus non-treated cells were performed in pairs of one treated and one non-treated coverslip within a time frame of 24 h.

### Western Blot Analysis

To determine the RNA interference efficiency in shSmn-transduced cells, the reduction in Smn protein was assessed by western blot. Total cell lysates were resolved on SDS-polyacrylamide gels and transferred onto polyvinylidene difluoride Immobilon-P transfer membrane filters (Millipore) using an Amersham Biosciences semidry Trans-Blot. The membranes were blotted with the anti-SMN antibody (1:5000; BD Biosciences). The membranes were reprobed with the monoclonal anti-α-tubulin antibody (1:50,000; Sigma), as a loading control. Blots were developed using Luminata™ Forte Western HRP Substrate (Millipore).

### Electrophysiology

To characterize the passive and active electrical properties of motoneurons in culture, the whole-cell configuration of the patch-clamp technique, in voltage-clamp and current-clamp modes, was used. Recordings were carried out at room temperature (RT, 22–24°C). Electrodes of 2–5 MΩ resistance were fabricated from borosilicate glass capillaries using a horizontal puller (Sutter Instrument Co., Model P-97). A silver chloride wire was used as a reference electrode. For current-clamp recordings, the pipette solution consisted of (in mM) 135 K-gluconate, 10 KCl, 9 NaCl, 1 MgCl_2_, 1 EGTA, 3 Mg-ATP, 0.3 Na_3_-GTP, and 10 HEPES, pH 7.35 (295–300 mOsm). The standard bath solution consisted of (mM) 140 NaCl, 5 KCl, 2.5 CaCl_2_, 1 MgCl_2_, 10 HEPES and 10 glucose, pH 7.4 (310–315 mOsm). To record sodium currents in isolation, the pipette solution contained (in mM) 140 Cs-methanesulfonate, 20 tetraethyl-ammonium (TEA)-Cl, 2 EGTA, 1 MgCl_2_, 0.2 CaCl_2_, 2 Mg-ATP and 10 HEPES, pH 7.3 with CsOH (295–300 mOsm). For these experiments, 2 mM 4-aminopyridine (4-AP) and 12 μM tetraethylammonium (TEA), and 0.2 mM CdCl_2_ were added to the standard bath solution to block potassium- and calcium-currents, respectively. All chemicals were purchased from Sigma-Aldrich. Motoneurons were visualized at 32× using an Axiovert 35 (Zeiss) microscope equipped with an epifluorescence system. Voltage-clamp and current-clamp recordings were obtained using a patch clamp amplifier EPC 10 (HEKA). Only cells with series resistances of <15 MΩ were accepted for analysis. Electronic compensation of series resistance was used when needed. Holding potential was −70 mV. Inward and outward current amplitudes during depolarization steps of 50 ms duration were measured at the peak and steady state, respectively, for each clamped voltage. Recordings with delayed activation of inward currents were discarded as a sign of inadequate clamp. For input resistance (R_in_) estimation, voltage changes in response to negative and positive current pulses of 250–500 ms duration were measured. R_in_ was then calculated as the slope of the linear voltage to current relationship in the range from −30 mV to +30 mV. All data were acquired and analyzed by using the Patchmaster software. The current density was calculated by dividing the peak amplitude of a given current by the membrane capacitance of the cell. Action potential amplitude was measured from the threshold baseline to the peak of the voltage deflection. Only green fluorescent (GFP^+^) motoneurons and GFP^+^ NSC-34 large cells with the neuron-like phenotype were recorded.

### Immunohistochemistry

Cultured motoneurons were fixed with 4% paraformaldehyde for 20 min (10 min on ice, 10 min at RT), permeabilized with 1% (v/v) Triton X-100 in PBS washing three times for 5 min each time. Cultures were incubated with the following primary antibodies: anti-bassoon (mouse monoclonal, 1:250, Enzo); anti-SMN (rabbit polyclonal, 1:250, Santa Cruz); anti-vGLUT2 (rabbit policlonal, 1:250, Synaptic Systems); anti-PSD-95 (mouse monoclonal, 1:250, Millipore); anti v-AChT (rabbit polyclonal, 1:250; Synaptic Systems) diluted in 5% (w/v) BSA, 1% Triton X-100 in PBS for 1 h. After washing three times for 5 min with 1% Triton X-100 in PBS, cells were incubated for 1 h with the secondary antibody (Alexa 594 or Alexa 647-conjugated donkey anti-rabbit or anti-mouse, Invitrogen) in PBS 1X containing 0.05% Triton X-100 (1:500) and washed with PBS three times for 5 min. Finally, cells were mounted with slowfade medium (Invitrogen) on microscope slides.

### Cell Imaging

Images were acquired with an upright Olympus FV1000 confocal microscope, equipped with three excitation laser lines (488 nm, 561 nm and 633 nm). An alternating sequence of laser pulses was used during the acquisition of images for sequential activation of the different fluorescent probes. A 60× oil immersion objective (N.A. = 1.42) was used. Images from EV- or shSmn-transduced or non-transduced cultured motoneurons were taken with similar conditions (laser intensity and photomultiplier voltage) on the same day. Fluorescence distribution and area of soma were analyzed using ImageJ routines. Motoneuron soma was delineated with outline masks based on brightness thresholding from maximal projected confocal images.

### Statistical Analysis

Statistical inferences were drawn from unpaired *t*-test or in the case of a non-normal distribution from Mann-Whitney test and presented as mean ± SEM. The results were considered as statistically significant with *p* < 0.05.

## Results

### Smn-Deficient Motoneurons Establish Fewer Synaptic Contacts than Control Motoneurons

**Figure 1:**
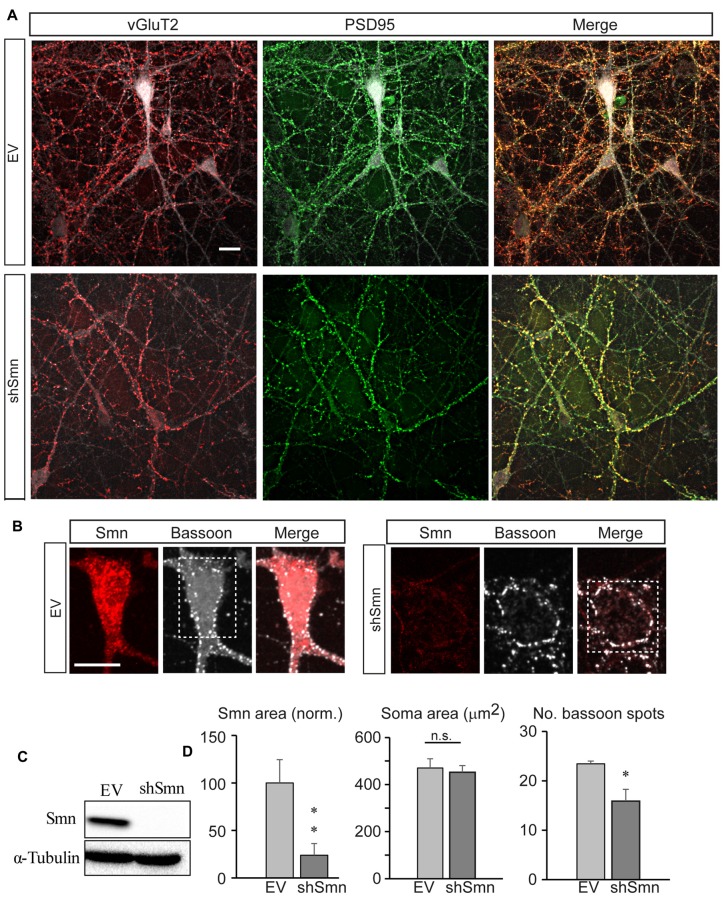
Smn-deficient spinal motoneurons in culture establish less synapse contacts than empty vector (EV)-transduced neurons. **(A)** Embryonic mouse motoneurons in primary culture develop an extended neural network with numerous synaptic contacts at DIV12, both in EV and shSmn-transduced cultures, as visualized by confocal microscopic Z-projections. Axosomatic and axodendritic synapses appear as punta (red, anti-vGLUT2; green, anti-PSD95. Merged images are also shown. **(B)** Examples of immunofluorescence for Smn (red) and bassoon (white) at the soma of EV and shSmn-transduced motoneurons. **(C)** Western blot for Smn content in EV and shSmn-transduced cultures. **(D)** Comparison of the Smn fluorescence surface area, somatic total surface area, and number of bassoon spots in EV and shSmn-transduced cultures. The Smn signal is significantly decreased in Smn-deficient motoneurons (*p* = 0.008. two-tail unpair *t*-test). The mean number of bassoon spots is also low in Smn-deficient motoneurons (16.1 ± 1.6 spots; 48 neurons, three independent experiments) in comparison with motoneurons in EV cultures (23.4 ± 2.1 spots; 31 neurons, 31 neurons, three independent experiments; *p = 0.006, two-tail unpair *t*-test). Error bars represent SEM. Calibration bars: 20 μm.

We used a previously developed *in vitro* model of SMA, i.e., the lentiviral RNA interference method to downregulate the Smn protein level in isolated mouse spinal cord motoneurons (Garcera et al., 2011). The transduction efficiency is excellent (GFP-positive cells) with significant Smn protein level reduction at 3 days of interference, which is maintained after 9 days (Garcera et al., 2011). Hence, we performed our experiments on day ten of interference. At that time, the purified motoneurons formed a complex network of long processes and established numerous synaptic contacts (Figure 1A). Although some boutons were immunoreactive for the vesicular acetylcholine transporter (vAChT, data not shown), a much larger number were positive for the vesicular glutamate transporter type 2 (vGluT2), confirming previous findings that motoneurons in culture form glutamatergic synapses with each other (Ullian et al., 2004). The vGluT2 positive puncta (Figure 1A, red) co-localized almost perfectly with postsynaptic density protein 95 (PSD95) spots (Figure 1A, green), as expected for the apposition of the pre- and postsynaptic elements, both in EV- (upper panels) and shSmn- (lower panels) transduced neurons (GFP positive). The efficacies of the lentiviral vector and the shRNA construction to knock-down Smn were confirmed both by western blot (Figure 1C) and by confocal microscopy (Figures 1B,D, red labeling). Quantification of the number of axo-somatic synaptic spots with an antibody against bassoon, a presynaptic active zone protein, revealed a significant 31.3% decrease in shSmn-transduced motoneurons (Figure 1D, right graph) compared with that in EV-transduced motoneurons (*p* < 0.006, three independent experiments). The somatic areas of motoneurons were not significantly different in both conditions (Figure 1D, middle graph). Our results are in agreement with a previous study that showed that Smn deficiency alters the intrinsic ability of motoneurons to form synapses *in vitro* (Zhou et al., 2016).

### Smn-Deficient Motoneurons Are Hyperexcitable and Display a Selective Increase in Sodium Current Density

**Figure 2:**
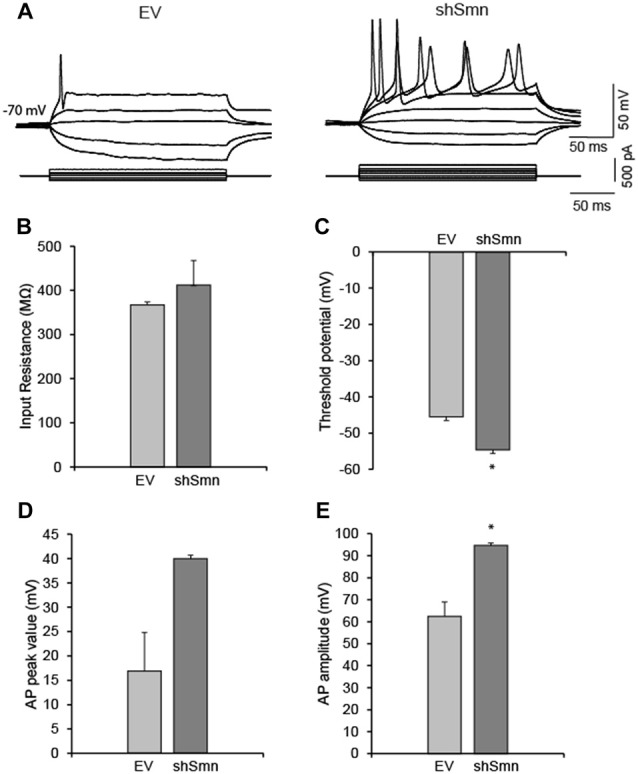
Smn-deficient motoneurons are hyperexcitable. **(A)** Examples of passive and active responses under current clamp in control (EV) and shSmn-transduced motoneurons. Upon current injection, Smn-deficient motoneurons exhibited a higher discharge rate than EV motoneurons. **(B)** The input resistance of the Smn-deficient motoneurons shows an upward trend compared with that in the EV-transduced motoneurons. **(C)** Smn-deficient motoneurons show a reduced action potential threshold potential relative to EV-transduced cells (*p* = 0.005). **(D,E)** shSmn-transduced motoneurons have larger action potential amplitudes compared to controls (*p* = 0.04). Resting potential was set at −70 mV by small holding currents. Graph values are the mean of the action potential amplitude for Smn-reduced and control motoneurons of three independent experiments ± SEM (error bars). Asterisk indicates significant differences between data from the experiments using student’s *t*-test (*p < 0.05).

We studied the passive and active membrane electrical properties of EV- and shSmn-transduced motoneurons in culture by using the whole-cell configuration of the patch clamp technique. Two examples of the respective responses to current injections are shown in Figure 2A. Stepwise depolarization for 500 ms typically produced a single action potential in EV neurons but repetitive firing in shSmn-transduced motoneurons. Although no significant difference in input resistance was evident (Figure 2B), in Smn-deficient motoneurons the first spike generated above rheobase presented a more negative action potential threshold (Figure 2C) and the spike peak potential was closer to the equilibrium potential of sodium ions (E_Na_, Figure 2D) than in controls, resulting in a significant larger action potential mean amplitude (Figure 2E). The shift in the action potential threshold towards more negative values increased the probability of firing in shSmn-transduced motoneurons.

**Figure 3:**
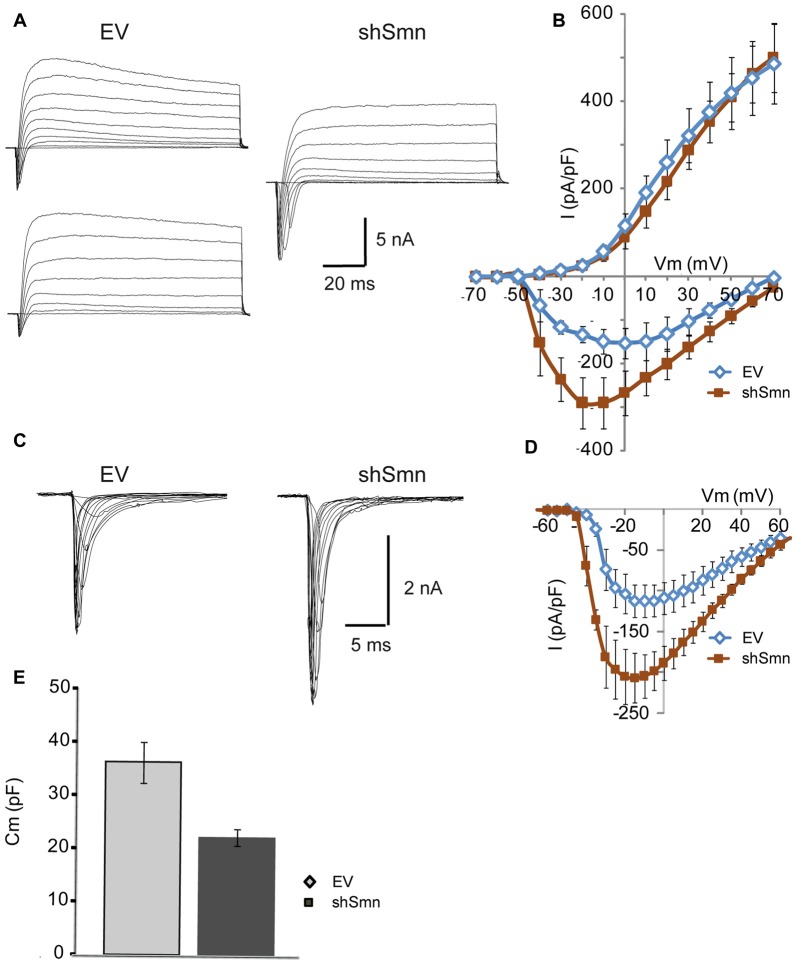
The sodium current density is increased in Smn-deficient motoneurons. **(A)** Examples of families of total whole-cell currents in EV and shSmn-transduced motoneurons. **(B)** I/V curves show an increase in inward current in Smn-deficient motoneurons (red symbols) compared to that in EV-transduced motoneurons (blue symbols). However, the size of the outward currents at the end of the pulse was not significantly different. Current amplitude was normalized to cell size by dividing the absolute value of the peak current by the membrane capacitance. **(C,D)** In the presence of potassium and calcium channel blockers, the peak sodium current was significantly larger in Smn-deficient cells than in EV-transduced cells. **(E)** Smn-deficient motoneurons have lower membrane electrical capacitance than control motoneurons (*p* = 0.03; *n* = 12).

To examine the mechanisms underlying the changes in the passive and active membrane properties described above, we performed whole-cell voltage-clamp experiments. In response to depolarizing steps from a holding potential of −70 mV, Smn-deficient motoneurons exhibited larger voltage-dependent macroscopic inward current densities than EV-transduced motoneurons, but no change in outward currents (Figures 3A,B). To isolate sodium current, we recorded in the presence of 2 mM 4-AP, 12 mM TEA, and 0.2 mM CdCl_2_ to block potassium and calcium currents, and replaced K^+^ in the internal pipette solution with Cs^+^. Representative sodium current traces in response to 50 ms depolarizing voltage steps from a holding potential of −70 mV are shown in Figure 3C. For a better comparison of the current density in each case, individual currents were normalized to the membrane capacitance of each cell. The smaller capacitance values in shSmn neurons compared to controls (Figure 3E) contrasts with the lack of soma size differences found after fixation (Figure 1C) but can be explained by the relatively low number of neurites in the mutant neurons. The current-voltage relations (Figure 3D), show that the mean peak current density increased by 83% in shSmn-transduced neurons (206.9 ± 30.3 pA/pF; *n* = 8) in comparison with EV-transduced motoneurons (113.1 ± 19.9 pA/pF; *n* = 7; *p* = 0.0023). On the other hand, we found no significant differences in the activation threshold in both cases, in accordance with a previous report in motoneurons differentiated from induced pluripotent stem cells (iPSCs) derived from fibroblasts of human SMA patients (Liu et al., 2015).

### Smn Deficiency Alters the Excitability of NSC-34 Cells before the Establishment of Synaptic Contacts

**Figure 4:**
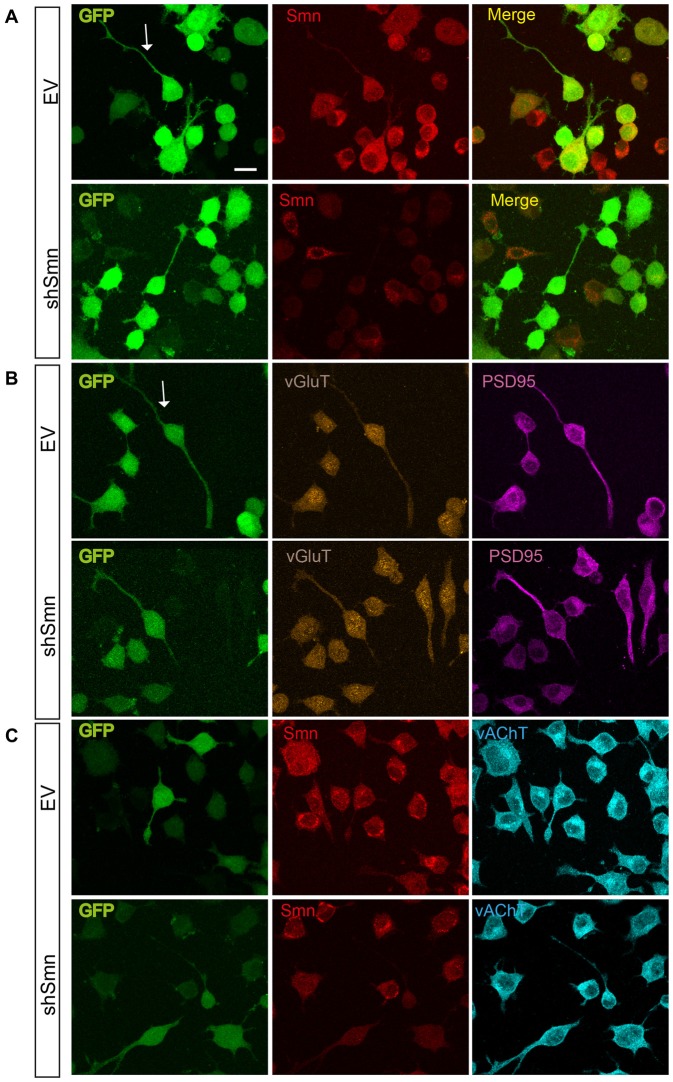
EV- and shSmn transduced NSC-34 neurons. Confocal images of fixed and immunostained control (EV) and shSmn cultures at DIV3 showing cells with bipolar or pyramidal somata, relatively long neurites and growth cone-like structures (examples marked by arrows). Lentivirally transduced cells display direct green fluorescence (GFP). **(A)** Representative examples of Smn expression as assessed by immunostaining in EV and shSmn-transduced cells (central panel). **(B,C)** Immunoreactivity to vGlut (**B**, central panels), PSD95 (**B**, right panels), and vesicular acetylcholine transporter (vAChT) (**C**, right panel) was homogeneous and revealed the absence of synaptic contacts. Calibration bars: 20 μm.

To explore whether Smn was able to regulate ion current densities in the absence of synaptic contacts, we used serum-deprived differentiated NSC-34 cells after 72 h in culture. NSC-34 is a spinal cord × neuroblastoma hybrid cell line, which expresses morphological and physiological properties of motoneuron cells. Lentiviral infection was performed as in the primary cultures of motoneurons. Three days after lentiviral interference, some cells presented a more developed differentiated motoneuron phenotype, typically with a bipolar or pyramidal aspect, neurites about 100 μm long, and growth cone-like structures (Figures 4A,B). No evident differences in neurite length or growth cone-like structures were observed between EV- and shSmn-transduced cultures. At this stage of the culture, synaptic connections between cells were not yet established. These were the cells used for electrophysiological recordings if they showed green fluorescence, indicative of GFP expression. To confirm that synaptic contacts were not established, immunolabeling against the vGluT2 and the postsynaptic protein PSD95 was performed in EV- and shSmn-transduced cultures. Fluorescence for both synaptic markers was homogeneous without puncta (Figures 4B,C), in contrast to primary motoneurons in culture (Figure 1A).

**Figure 5:**
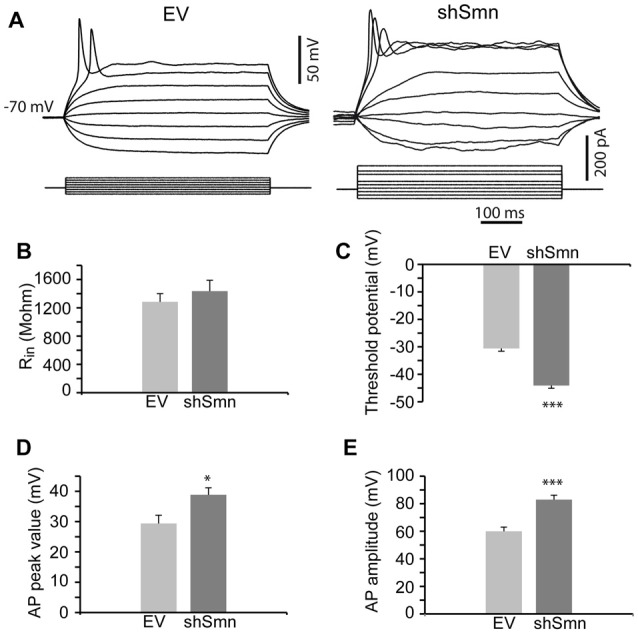
Smn-deficient NSC-34 cells are hyperexcitable. **(A)** Examples of passive and active responses under current clamp in EV and shSmn-transduced cells. **(B)** The mean input resistance of the Smn-deficient cells was not significantly different from EV-transduced cells. **(C)** Smn-deficient cells show a reduced action potential threshold potential relative to EV-transduced cells (*p* < 0.0001; *n* = 24 and 29 cells). **(D,E)** shSmn-transduced cells have larger action potential amplitude compared to control motoneurons (*p* < 0.0001). Resting potential was set at −70 mV by small holding currents. Graph values are the mean of the action potential amplitude for Smn-reduced and control cells of three independent experiments ± SEM (error bars). Asterisk indicates significant differences between data from the experiments using student’s *t*-test (*p < 0.05; ***p < 0.0005).

Reduced Smn expression in NSC-34 cells modified active (but not passive) electrical properties of the neurons. For example, the membrane input resistance did not change (Figure 5B), but the action potential threshold shifted to potentials more negative (Figure 5C, *p* < 0.0001; *n* = 24 and 29 cells), increasing the probability of producing action potentials. Concurrently, the inversion peak potential (Figure 5D, *p* = 0.01) and the amplitude of the action potential (Figure 5E, *p* < 0.0001; *n* = 24 and 29 cells) increased in shSmn-transduced neurons. Nevertheless, contrary to that what occurs during normal development in motoneurons, these changes were not accompanied by an increase in the afterhyperpolarizing potential. In general, only one action potential could be elicited during intracellular injection of prolonged depolarizing currents in control and Smn-depleted cells (Figure 5A), although in some cases afterdepolarization potentials were evidenced in shSmn transduced cells.

### Smn-Depletion Increases the Density of the Sodium Current, but Not the Potassium Current of NSC-34 Cells

**Figure 6:**
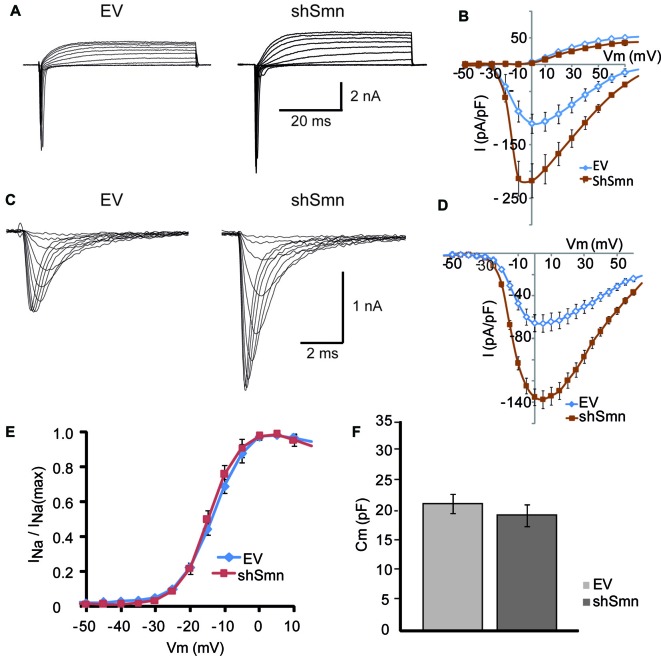
The sodium current density is increased in Smn-deficient NSC-34 cells. **(A)** Examples of families of total whole-cell currents in EV and shSmn-transduced NSC-34 cells. **(B)** I/V curves show an increase in inward current in Smn-deficient neurons (red symbols) compared to that in EV-transduced neurons (blue symbols). However, the size of the outward currents at the end of the pulse was not significantly different. Current amplitude was normalized to cell size by dividing the absolute value of the peak current by the membrane capacitance. **(C,D)** In the presence of potassium and calcium channel blockers, the peak sodium current was significantly larger in Smn-deficient than in EV cells. **(E)** Voltage-dependence of the sodium current in control and Smn-deficient cells were not significantly different. **(F)** In Smn-deficient cells membrane electrical capacitance was similar to control (*p* = 0.03; *n* = 18).

Using whole-cell voltage clamp, we elicited inward and outward currents by depolarizing steps from a holding potential of −70 mV. In NSC-34 cells, the space clamp was better than in embryonic motoneuron primary cultures, probably due to the shorter length of their neurites. Individual current amplitudes were divided by cell membrane capacitance (Figure 6F) to obtain the density of membrane current. As in primary motoneuron cultures, inward currents, but not outward currents, were increased in Smn-deficient cells, compared to controls (Figures 6A,B). The small amplitude of the potassium currents in NSC-34 cells may explain the lack of repetitive firing, even in Smn-depleted cells (Figure 5A).

Sodium currents were recorded in isolation by blocking the potassium and calcium conductances by extracellular blockers and ion substitution (see “Materials and Methods” Section). The peak sodium current was increased two-fold in shSmn-transduced cells (to 137.5 ± 18.8 pA/pF; *n* = 3) compared with controls (65.8 ± 8.9 pA/pF; *n* = 4; Figures 6C,D). We next investigated whether the activation voltage-dependence of the sodium channels was changed between the two conditions by normalizing the peak current at each voltage to the maximum peak current, and found no significant difference between control and Smn-deficient cells (Figure 6E). These data show that Smn-deficiency increases the density of voltage-dependent sodium channels in NSC-34 cells without altering their activation properties.

### Glutamate Blockers do Not Change Current Densities in NSC-34 Cells

**Figure 7:**
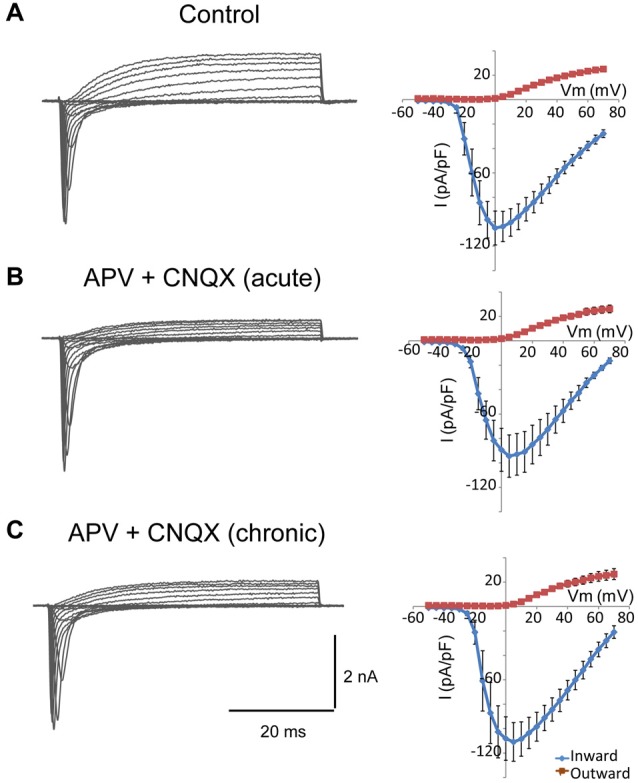
Glutamate receptor blockers do not change the density of sodium and potassium currents in NSC-34 cells.** (A–C)** Examples of families of total whole-cell currents in NSC-34 cells (left) and I/V curves show no significant differences in outward or inward current amplitudes among cells recorded in the absence of drugs **(A)**; *n* = 13, in the presence of APV (100 μM) + CNQX (20 μM) during the recording **(B)**; *n* = 10, and incubated for 3 days with the glutamate blockers **(C)**; *n* = 11. Current amplitudes were normalized to cell size by dividing the absolute value of the peak current by the membrane capacitance.

In cultured neurons, chronic pharmacological blockade of the spontaneous electrical activity with tetrodotoxin, or inhibition of glutamate receptor activation with specific blockers, lowers the action potential firing threshold and increases the sodium current density (Desai et al., 1999). Differentiated NSC-34 cells are responsive to glutamate and express glutamate receptors (Eggett et al., 2000). Thus, we wondered whether the increase in the sodium current density observed in Smn-depleted NSC-34 cells could be due to a reduction in the paracrine and autocrine secretion of glutamate in the absence of synaptic contacts. To investigate this possibility, we chronically treated the cultures with specific glutamate receptor blockers, APV (100 μM) and CNQX (μM), and compared the electrophysiological properties of the cells in the presence or not of the drugs. As an additional control, we tested the effect of the acute application of glutamate blockers only during the recordings and confirmed that they had no effect on the sodium and potassium currents elicited by voltage steps (Figure 7B). The cells exposed for 3 days to the drugs showed no changes in cell morphology, action potential production, or ion current densities (Figure 7C) as compared to cultures without blockers (Figure 7A). The results further support the conclusion that the changes in cellular excitability in Smn-depleted NSC-34 cells were not influenced by glutamate excitation.

## Discussion

Homeostatic plasticity adjusts the activity of the neural networks to variable physiological states, including those caused by pathological processes. Homeostasis is achieved by changes in both synaptic strength and the intrinsic excitability of neurons. Currently, a large amount of information is available about synaptic plasticity, but much less is known about the physiological mechanisms involved in the intrinsic regulation of the passive and active electrical membrane properties of neurons. Here, we investigated the changes in membrane excitability in two cellular models of SMA, one in which the motoneurons are integrated into a network with a lowered number of synaptic inputs, and another one in which neurons established no synaptic contacts. These approaches allowed us to determine if Smn-deficiency changes cell excitability intrinsically. Our results show that hyperexcitability is mediated by a selective rise in voltage-dependent sodium current density, which can occur independently of the establishment of synaptic contacts.

### Synaptic Contacts and Motoneuron Hyperexcitability

Normally, activity-deprived neurons respond by lowering action potential threshold and increasing sodium current density, which provides a means of ensuring effective action potential generation and neurotransmitter release (Desai, 2003). Recent studies have shown that SMA motoneurons exhibit hyperexcitability consisting of high input resistance, low activation threshold, APs of large amplitude and fast rising rate, and high action potential firing frequency (Mentis et al., 2011; Liu et al., 2015). Concomitantly, SMA motoneurons have a reduced number of excitatory synaptic inputs (Mentis et al., 2011; Ling et al., 2012; Simon et al., 2016), together with defective neurotransmitter release at motor nerve terminals (Kong et al., 2009; Ruiz et al., 2010; Torres-Benito et al., 2011; Ruiz and Tabares, 2014; Tejero et al., 2016). Hence, these deficits could contribute to hyperexcitability.

The origin of the reduced number of synaptic inputs onto SMA motoneurons is under debate. Possible mechanisms include the primary deficit of Smn in motoneurons and neuronal and non-neuronal cells. Experimentally, when Smn is depleted only in motoneurons, peripheral synaptic defects appear (Park et al., 2010), and when Smn is restored only in motoneurons of an SMA mouse model, somatic synaptic inputs are restored (Gogliotti et al., 2012), suggesting a cell-autonomous mechanism in motoneurons. In line with these results, in our experiments with purified motoneuron in cultures, the number of synaptic contacts was lower in Smn-depleted than in control motoneurons, indicating that the intrinsic ability of motoneurons to form synapses *in vitro* is altered. On the other hand, in a series of coculture experiments, it has been reported that Smn deficiency only in interneurons also leads to a loss of somatic excitatory synapses onto wild-type motoneurons, although less than when Smn is reduced in both cell types (Simon et al., 2016). Similarly, when motoneurons were cocultured with astrocytes, synapse formation was significantly reduced when either motoneurons, astrocytes or both were Smn defective (Zhou et al., 2016). Together, these data reveal the importance of Smn for synapse formation and maintenance in motoneurons, proprioceptive sensory neurons and interneurons.

### Changes in the Passive and Active Properties of the Membrane by Smn-Deficiency

The passive properties of the membrane (capacitance and resistance) shape the postsynaptic electrical response, while the active properties of the membrane (number and type of voltage-dependent ion channels) determine the characteristics of the action potential. An increase in membrane input resistance reduces the action potential threshold for a given synaptic drive. In SMA mouse models, motoneuron membrane input resistance has been shown to be increased *in vivo* with respect to controls (Mentis et al., 2011; Gogliotti et al., 2012; Zhou et al., 2016; Fletcher et al., 2017), but different results have been reported *in vitro*. In purified motoneuron culture, both an increase (Liu et al., 2015) and no significant change (Simon et al., 2016; Zhou et al., 2016) in input resistance between control and Smn-deficient motoneurons have been reported.

Given that in our experiments the membrane input resistance was not significantly different in Smn-depleted motoneurons with respect to controls, the signs of hyperexcitability displayed in these neurons, i.e., low action potential activation threshold, APs of large amplitude and fast-rising rate, and increase in firing frequency, are likely due to the change in voltage-dependent sodium current density. The increase in sodium current was selective as did not affect the size of the potassium current. Similar results were obtained in the absence of synaptic contacts in NSC-34 motoneuron-like cells, suggesting that membrane input resistance and sodium current density can be independently regulated. In accordance with our results, it has been shown that in SMA patient-derived motoneurons in culture the density of sodium current is also larger than in control motoneurons while the potassium current does not change (Liu et al., 2015). However, a reduction of Kv2.1 channel expression in SMA motoneurons, driven by the loss of proprioceptive synaptic inputs, has been reported in neonatal spinal cord preparations (Fletcher et al., 2017). This finding is of interest because points to a molecular mechanism by which the sensory synaptic network can change motoneurons excitability.

### Mechanisms of Sodium Current Increase in Smn-Depleted Neurons

Our results show that changes in the voltage-dependent sodium conductance density contribute to the intrinsic responsiveness of Smn-defective motoneurons before the establishment of synaptic contacts. To examine the mechanism by which this occurs, we investigated a potential paracrine and autocrine effect of glutamate released by growth cones (Young and Poo, 1983) and axons (Zakharenko et al., 1999), under the hypothesis that in Smn-depleted cells the secretion of glutamate was less than in controls. However, this possibility can be discarded as no increase in sodium current was elicited after incubation of control cells with blockers of AMPA and NMDA receptors. Other possibilities are that the reduction in the Smn level changed the concentration of other secreted diffusible factors, or their respective membrane receptors, in both culture models. For example, it has been shown that growth factors such as nerve growth factor (NGF) and Fibroblast growth factors (FGF) regulate the expression of voltage-dependent sodium channels (D’Arcangelo et al., 1993). Finally, although Smn level gradually decreases during development (La Bella et al., 1998; Jablonka et al., 2001) and the density of the voltage-dependent sodium channel follows the opposite trajectory (Gao and Ziskind-Conhaim, 1998), a direct or indirect, cell-autonomous, effect of Smn level on the regulation of voltage-dependent sodium channel expression has not been demonstrated.

### Relationship between Hyperexcitability and Cell Damage

A key question still unresolved is whether hyperexcitability could affect Smn-deficient motoneuron wellness and survival. Although motoneuron death can occur independently of hyperexcitability (Simon et al., 2016), it might be that heightened electrical activity contributes importantly to motoneuron damage in SMA; for example, by excessive calcium entry during repetitive action potential firing, or by deficient buffering of cytosolic calcium upon stimulation. Recently, we have shown that the density of P/Q-type voltage-dependent calcium channels is significantly reduced in motor nerve terminals of SMA mutant mice (Tejero et al., 2016), which makes it unlikely that excessive calcium entry occurs during evoked release. However, we also have found an increase in asynchronous release in severely affected nerve terminals (Ruiz et al., 2010), which could result from an insufficient buffering of cytosolic calcium. Mitochondrial density is significantly lower in SMA than in control presynaptic motor terminals (Torres-Benito et al., 2011), and mitochondrial dysfunction in SMA motoneurons has been documented recently (Miller et al., 2016). One of the roles of mitochondria is the regulation of intraterminal cytosolic calcium levels, providing an especially important buffering action during repetitive action potential firing. Therefore, a low number of mitochondria, an alteration in their spatial distribution, or an impairment of their functional capacity may interfere with calcium buffering, producing toxic calcium overloads. Remarkably, in human amyotrophic lateral sclerosis (ALS) patients, presymptomatic cortical hyperexcitability has been described (Vucic and Kiernan, 2008) and cultured spinal and cortical motoneurons from ALS mice are hyperexcitable (Kuo et al., 2004, 2005; Saba et al., 2016), with no alteration of passive membrane properties (Pieri et al., 2003; Kuo et al., 2004), together with altered mitochondrial and energy metabolism (Heath and Shaw, 2002; Ellis et al., 2003). The hyperexcitability of motoneurons in the ALS G93A mouse model has been attributed to an increase in sodium current (Pieri et al., 2003; Kuo et al., 2004, 2005).

## Conclusion

Our results suggest that SMN levels regulate neuronal excitability, both before and after synaptogenesis. The fact that SMN may regulate sodium channel density at an early stage of motoneuron development has not been reported before. Collectively, the results indicate that the pathological decrease of SMN triggers the activation of cell-autonomous and non-cell autonomous pathways and that the interplay between synaptic inputs and intrinsic excitability regulates the sensorimotor neural network activity as a whole.

## Author Contributions

SA: conducted the experiments, analyzed and interpreted the results. AG and RMS: designed and fabricated the constructions and analyzed the results. LT: designed the experiments, analyzed, interpreted the results and wrote the manuscript.

## Conflict of Interest Statement

The authors declare that the research was conducted in the absence of any commercial or financial relationships that could be construed as a potential conflict of interest.

## Abbreviations

4-AP: 4-aminopyridine
ACh: Acetylcholine
ALS: Amyotrophic lateral sclerosis
APV: DL(-)-2-amino-5-phosphonopentanoic acid
CNQX: 6-cyano-7-nitroquinoxaline-2,3-dione
EV: Empty Vector
FGF: Fibroblast growth factors
GFP: Green fluorescence protein
iPSCs: induced Pluripotent stem cells
NGF: Nerve growth factor
NMJ: Neuromuscular junction
PSD95: Postsynaptic density protein 95
shRNA: short hairpin RNA
SMA: Spinal muscular atrophy
SMN: Survival of Motor Neuron
TEA: Tetraethylammonium
vAChT: Vesicular acetylcholine transporter
svGluT2: vesicular glutamate transporter type 2.
